# Atypical Overt Hemorrhagic Stroke in an Adult With Sickle Cell-Hemoglobin C Disease: A Report of a Rare Case

**DOI:** 10.7759/cureus.100573

**Published:** 2026-01-01

**Authors:** Nikita S Manjrekar, Alexander M Preisig, Chaithanya Singh, Montasin Rezwan

**Affiliations:** 1 Department of Medicine, William Carey University College of Osteopathic Medicine, Hattiesburg, USA; 2 Department of Medicine, Ross University School of Medicine, Bridgetown, BRB; 3 Department of Family Medicine, St. John's Episcopal Hospital, Far Rockaway, USA; 4 Department of Internal Medicine, St. John's Episcopal Hospital, Far Rockaway, USA

**Keywords:** cerebrovascular accident (stroke), clinical hematology, hemoglobinopathy, hemoglobin sc disease, hemorrhagic stroke

## Abstract

Sickle cell disease (SCD) is an inherited hemoglobinopathy in which deformed erythrocytes impair microvascular blood flow, leading to complications across multiple organ systems. Variants of SCD exist, and a less common form involves the inheritance of one hemoglobin S allele and one hemoglobin C allele, manifesting as sickle cell-hemoglobin C (HbSC) disease. Although HbSC disease can cause significant sequelae, a rare but serious complication is hemorrhagic stroke. In such cases, chronic endothelial injury, vascular fragility, and abnormal hemodynamics associated with this hemoglobinopathy may predispose patients to vessel rupture, resulting in hemorrhage. We report the case of a 54-year-old man with HbSC disease who was admitted for an acute sickle cell crisis and subsequently experienced an overt hemorrhagic stroke. The patient developed chronic neurological deficits and remains under skilled nursing care. This case highlights the rare occurrence of hemorrhagic stroke in HbSC disease and challenges the perception that HbSC is a benign condition.

## Introduction

Sickle cell disease (SCD) is a hereditary hematologic disorder in which the inability to produce normal β-globin chains leads to hemoglobin molecules that polymerize under certain environmental conditions. Consequently, erythrocytes assume a crescent or “sickled” shape [1]. The most common variant of SCD, widely discussed in the literature, is the homozygous HbSS condition. A less common variant, with more limited literature coverage, is HbSC, in which patients inherit one HbS gene and one HbC gene [2]. The HbS mutation results in the substitution of valine for glutamate at the sixth position of the β-globin chain, whereas the HbC mutation substitutes lysine for glutamate at the same position [1].

Although classified under SCD, HbSC disease has a distinct pathophysiology due to the presence of HbC, resulting in a unique spectrum of clinical severity and complications. One of the most significant complications is stroke. Studies indicate that patients with HbSC disease are more likely to experience ischemic rather than hemorrhagic strokes and are more prone to silent strokes than overt strokes [3,4].

Our patient experienced an overt hemorrhagic stroke, an uncommon occurrence in HbSC disease. Because hemorrhagic stroke is rarely documented in this population, this case contributes to the limited evidence base and underscores the need for heightened clinical awareness. The case presentation details the patient’s clinical course and rapid deterioration, emphasizing the rarity of this presentation.

## Case presentation

A 54-year-old African-American male with sickle cell-hemoglobin C (HbSC) disease presented to the emergency department of our community hospital with bilateral paralumbar back pain unrelieved by his home regimen of acetaminophen. His past medical history was notable for hypertension, for which he was taking appropriately dosed daily medications. The trigger for his pain was unclear. His only reported SCD complication was occasional vaso-occlusive crises, managed with acetaminophen; he had never received disease-modifying therapy and had no hematology follow-up.

He denied fevers, chills, headaches, cough, dyspnea, hemoptysis, hematemesis, hematochezia, or any history of splenectomy or cholelithiasis. He had no known drug allergies and denied tobacco, alcohol, or illicit drug use. On presentation, vital signs were as follows: temperature 98.2 °F, blood pressure 173/94 mmHg, heart rate 79 beats per minute, respiratory rate 18 breaths per minute, and oxygen saturation 96%.

A chest radiograph showed no acute findings and was considered noncontributory to the neurological aspects of this case. Physical examination was unremarkable, including a normal neurologic assessment with no deficits or weakness.

Laboratory evaluation revealed hemoglobin 10.7 g/dL (reference range: 13.5-17.5), hematocrit 29.4% (41-53), reticulocyte count 2.2% (0.5-1.5), 3+ target cells, 2+ stomatocytes, and no sickle cells. Serum testing showed lactate dehydrogenase 258 U/L (50-242), total bilirubin 1.6 mg/dL (0.1-1.0), and albumin 5.2 g/dL (3.5-5.0) (Table 1). His diagnosis of HbSC disease had been previously established at another facility.

**Table 1:** Relevant laboratory results ALT, alanine aminotransferase; AST, aspartate aminotransferase

Laboratory test	Result	Normal range
Hemoglobin (g/dL)	10.7	12-16 (female), 13.5-17.5 (male)
Hematocrit (%)	29.4	36-46% (female), 41-53% (male)
WBC count (K/µL)	6.6	4.0-11.0
Platelet count (K/µL)	112	150-400
Reticulocyte count (%)	2.2	0.5-2.5%
Total bilirubin (mg/dL)	1.6	0.1-1.2
Lipase	1240	10-140 U/L
Albumin (g/dL)	5.2	3.5-5.0
Glucose	127	70-99
Creatinine (mg/dL)	1	0.6-1.1 (female), 0.7-1.3 (male)
AST (U/L)	28	10-40
ALT (U/L)	14	7-56
Urinalysis - protein	Negative	Negative
Urinalysis - blood	Negative	Negative
Urine glucose	Trace (100 mg/dL)	<30 mg/g

He was admitted to the inpatient unit with a diagnosis of acute sickle cell crisis. His treatment included intravenous fluid resuscitation, pain management, blood pressure control, and serial serum monitoring. He was stabilized and observed every 15 minutes, maintaining a National Institutes of Health Stroke Scale score of 0 [5].

However, just under 24 hours after admission, he experienced an acute change in his clinical status and became unresponsive. At that time, his blood pressure was 189/102 mmHg, and his heart rate was 110 bpm. Neurological examination documented multiple episodes of emesis, altered mentation, nonverbal status, and inability to follow commands. Profound weakness in the right upper and lower extremities with decreased sensation was noted.

**Figure 1:**
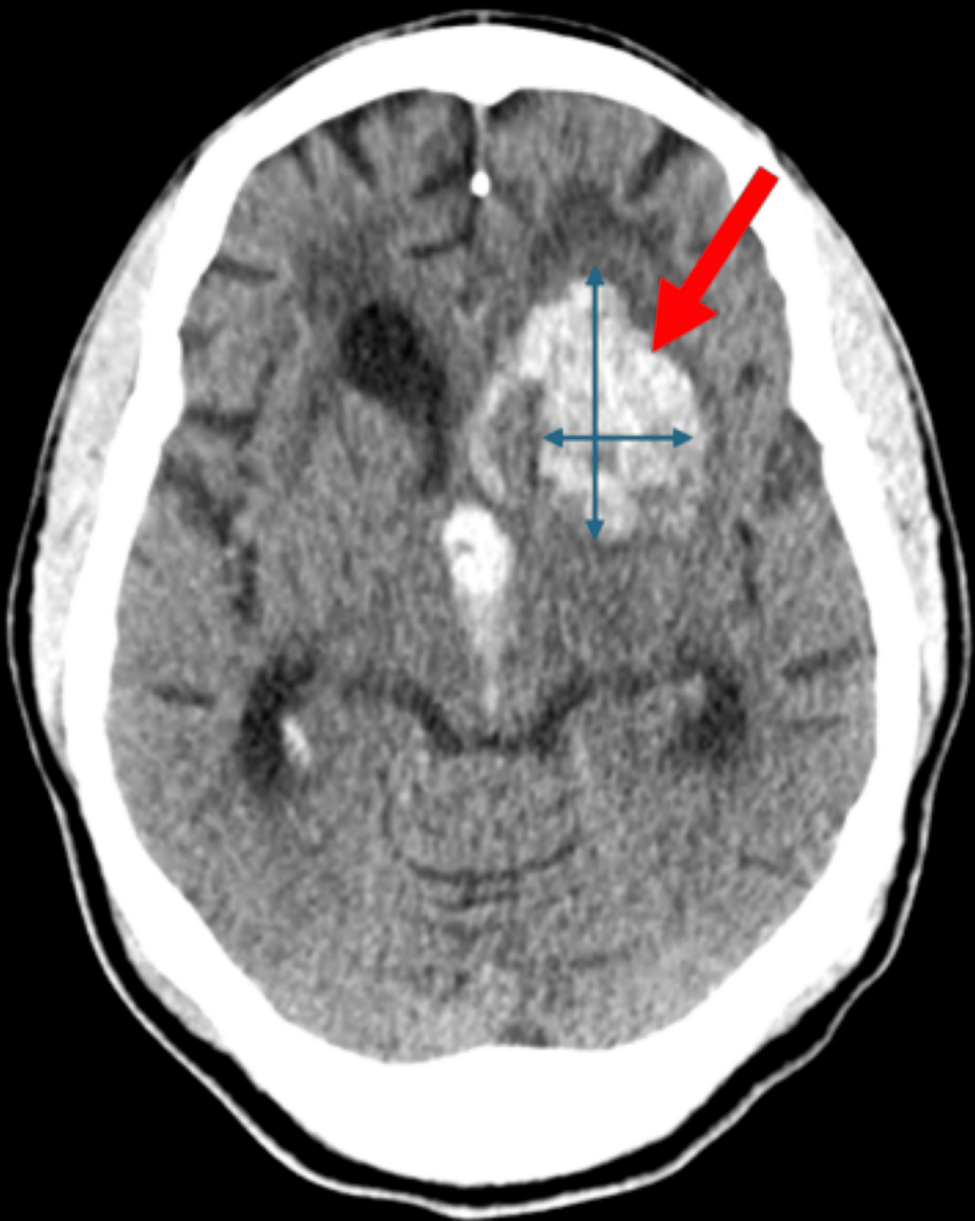
Axial non-contrast CT showing a left gangliocapsular intracerebral hemorrhage with surrounding edema and an associated rightward midline shift

A CT scan of the head revealed a 3.0 × 4.6 cm left gangliocapsular acute intraparenchymal hemorrhage with surrounding edema and localized mass effect (Figure 1). Moderate intraventricular extension of the hemorrhage was present, along with a 0.6 cm left-to-right midline shift. Extensive chronic small vessel ischemic changes of the cerebral white matter bilaterally and mild ventriculomegaly were also observed.

The patient underwent rapid sequence intubation and was subsequently transferred to a neurological intensive care unit at an outside facility. There, he underwent placement of an external ventricular drain, percutaneous endoscopic gastrostomy tube placement, and exchange transfusion with 10 units of packed red blood cells.

The diagnosis of hemoglobin SC disease was confirmed by hemoglobin electrophoresis performed at the tertiary center to which he was transferred for management of the intracerebral hemorrhage. Although the detailed electrophoresis report was not available in our medical record, confirmation was obtained through direct communication with the receiving facility.

During a subsequent hospitalization for an unrelated reason, the patient was noted to have survived the hemorrhagic stroke but exhibited significant neurological deficits. He had no verbal output or ability to follow commands; his eyes were midline with movement only to the left; he had a right facial droop, increased tone in the right upper extremity, minimal movement in the left upper extremity, no movement in the lower extremities, and decreased sensation. He requires ongoing skilled nursing care.

## Discussion

HbSC disease is the second most common variant of SCD after HbSS. Patients with HbSC are double heterozygotes, and their erythrocytes have higher intracellular hemoglobin concentrations, making them more susceptible to dehydration. Although HbSC occurs in approximately one in every 7,100 births in the United States, it remains underrepresented in the literature, partly because it has historically been considered a milder clinical phenotype. This perception is reflected in the relatively low use of disease-modifying therapies in this population. In contrast, hydroxyurea is the first-line therapy for HbSS [6]. Its use in HbSC is less frequent due to limited clinical trial data, which has hindered the development of formal treatment guidelines [2].

As with other SCD variants, complications in HbSC arise from repeated erythrocyte sickling in the microvasculature [2,7]. In HbSS, sickling occurs preferentially in smaller capillaries due to decreased transit time, further impairing blood flow and precipitating vaso-occlusive crises [7]. Over time, erythrocytes accumulate membrane damage, rendering their sickled morphology permanent, even under adequate oxygenation [1,7].

HbSC introduces additional considerations because each erythrocyte contains roughly 50% HbS and 50% HbC [2]. The presence of HbC promotes dehydration by enhancing potassium efflux and water loss [8]. This environment favors HbS polymerization, leading to hyperhemolysis, microvascular obstruction, and diffuse vasculopathy [3]. Despite sharing pathophysiological mechanisms with HbSS, HbSC is generally considered less severe, with lower incidences of sepsis and stroke. Elevated nitric oxide levels in these patients may further mitigate inflammation and cell adhesion. Nonetheless, HbSC carries its own spectrum of complications, including retinopathy, avascular necrosis, acute chest syndrome, splenomegaly, and depression. One of the most severe, albeit rare, complications is stroke [2].

Strokes in HbSC patients can be ischemic or hemorrhagic. Both types are more common in HbSS and are well-described in the literature [8]. In HbSC, vascular complications, including stroke, have a much lower prevalence [3]. Nelson et al. reported that while both ischemic and hemorrhagic strokes occurred in their HbSC cohort, the odds ratio for hemorrhagic stroke was not statistically significant [2]. Consequently, the development of overt hemorrhagic strokes in HbSC is exceedingly rare [2].

Strokes are also categorized as silent or overt [9]. Overt strokes present with clinically apparent signs, whereas silent strokes are subclinical [4]. In our patient, an overt hemorrhagic stroke occurred during a refractory pain crisis. The incidence of overt strokes in HbSC has been reported as negligible: Guilliams et al. and Pegelow et al. identified no overt strokes in their cohorts, while Saini et al. reported a single case [9-11]. Our patient’s older age falls outside the range typically studied in existing literature. Nevertheless, silent strokes remain more common than overt strokes in this population.

The precise etiology of the patient’s hemorrhagic stroke is unclear. However, contributing factors likely included extensive chronic small-vessel ischemic changes on CT [12,13], the underlying hemoglobinopathy [3], history of severe pain crises, and advanced age, which together may have acted synergistically to precipitate the event.

Although HbSC patients are generally at lower risk for severe neurological complications than HbSS, this case demonstrates that significant cerebrovascular events can occur [4]. Clinicians should consider close neurological monitoring during acute crises in high-risk patients. Disease-modifying therapies, such as hydroxyurea or transfusion programs, may be considered on an individual basis, though evidence for HbSC remains limited.

## Conclusions

This case highlights a rare but clinically significant event: an overt hemorrhagic stroke in a patient with HbSC disease. Key clinical features, including profound neurological deficits, and imaging findings, including a left gangliocapsular intracerebral hemorrhage with mass effect, underscore the potential for severe cerebrovascular complications even in this relatively milder sickle cell genotype. By documenting this uncommon presentation, this report contributes to the understanding of cerebrovascular risk in HbSC and emphasizes the importance of vigilance for neurological changes. These findings support individualized monitoring and prompt evaluation during acute crises to optimize management and outcomes.
